# Severe postoperative negative pressure pulmonary edema: a case report

**DOI:** 10.1186/s12871-024-02785-2

**Published:** 2024-11-01

**Authors:** Philipp Kazuo Omuro, David Sander, Dominique Hart

**Affiliations:** Department of Anesthesiology and Operative Intensive Care Medicine, University Hospital of Cologne, Kerpener Str. 62, Cologne, 50937 Germany

**Keywords:** Laryngospasm, Pulmonary edema, Intraoperative complications, ARDS, APRV Ventilation Mode

## Abstract

**Background:**

Postoperative negative pressure pulmonary edema (NPPE) can occur in any patient undergoing general anesthesia. There are several risk factors for it, especially postoperative laryngospasm. The disease is usually benign and quickly reversible. In our case the severity and need for advanced critical care therapy was unusual.

**Case:**

We report a severe case of postoperative negative pressure pulmonary edema in a 62-year-old male patient undergoing elective right-sided retroperitoneoscopic adrenalectomy. The patient developed a severe case of acute respiratory distress syndrome (ARDS) after postoperative laryngospasm, possibly in conjunction with a suspected anaphylactic reaction. The patient was consequently treated with a combination of invasive airway pressure release ventilation (APRV) and a prone positioning regimen. After drastic improvement in respiratory function, the patient was discharged from the intensive care unit after 10 days and from the hospital after 14 days.

**Conclusion:**

NPPE is a rare but relevant complication of anesthesia and laryngospasm. The disease can basically occur in any patient undergoing general anesthesia and therefore should be considered.

## Case

We report the case of a 62-year-old male undergoing elective right- sided retroperitoneoscopic adrenalectomy due to pheochromocytoma. The patient had a bodyweight of 75 kg and a body height of 175 cm. Arterial hypertension was the only finding in his medical history. Previously, he underwent uneventful general anesthesia twice. A preoperative treatment of the pheochromocytoma-induced mild arterial hypertension by alpha receptor blockade was not recommended by the endocrinologists. In the pre-anesthesia work up no signs of difficulty airway was detected (Mallampati I, mouth opening > 5 cm and neck mobility).

Following the uneventful induction of anesthesia by intravenous (i.v.) application of 30 µg Sufentanil, 400 mg Propofol and 50 mg Rocuronium bougie-guided endotracheal intubation was performed in Cormack & Lehane III conditions upon direct laryngoscopy. Balanced general anesthesia was maintained by Sevoflurane inhalation (end-expiratory concentrations 1,6 − 1,8 Vol%) and continuous i.v. infusion of Remifentanil (0,3 µg/kg/min). Intraoperative arterial hypotension necessitated hemodynamic support employing Noradrenaline at i.v. doses up to 0,3 µg/kg/min. An unexplained development of facial exanthema led to the clinical diagnosis of an allergic reaction of unknown cause which was subsequently treated by administration of 250 mg Prednisolone i.v. and 8 mg Dimetindene. There were no signs of accompanied hemodynamic instability or bronchospasm. Upon termination of surgery neuromuscular blockade was reversed by 200 mg Sugammadex i.v. and a Train-of-Four count (TOF) of 100% was achieved.

Following extubation the patient immediately developed clinical signs of a severe case of laryngospasm (inspiratory stridor, orthopnea and hyperventilation) resulting in a dip in the peripheral oxygen saturation (SpO_2_) to 88%. In response to lacking a patent airway due to laryngospasm and the imminent threat of hypoxia, general anesthesia was reinstituted using propofol and remifentanil. The airway was secured via laryngeal mask placement, thus reestablishing controlled ventilation and oxygenation. After the return of sufficient spontaneous breathing with adequate oxygenation anesthesia was terminated and the laryngeal mask was removed.

**Fig. 1:**
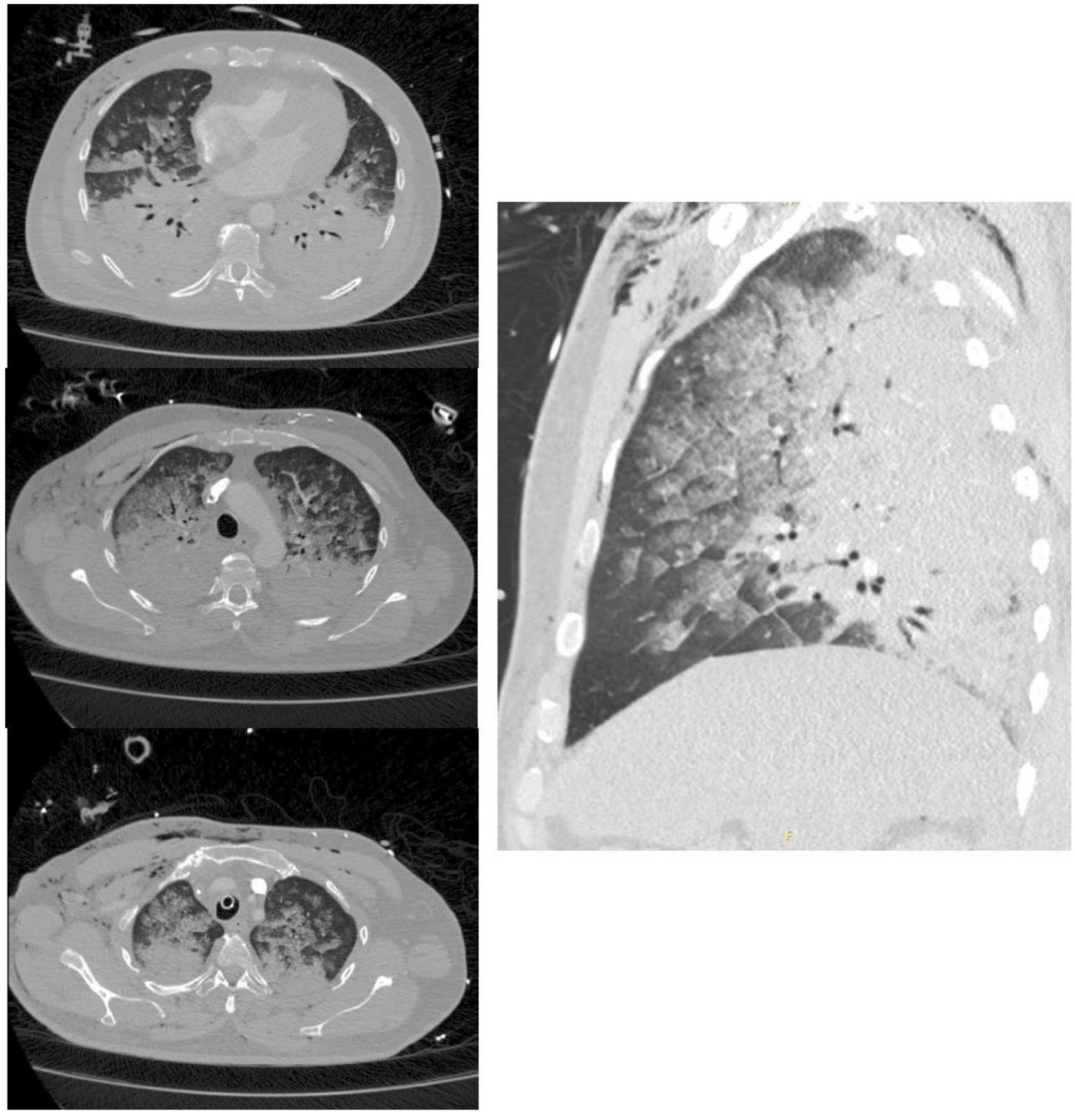
CT-scan with angiography of the thorax. Approximately 20 min post-intubution on postoperative day 0. Three transversal views from supradiaphragmal to apical (left). One sagittal view (right). Presence of severe alveolar interstitial bi-pulmonary edema. No signs of pulmonary artery embolism. Postoperative ventral subcutaneous emphysema

During the transfer from the operation room to the post-anesthesia care unit the patient developed a rapidly deteriorating hypoxia with a decrease in SpO_2_ to 65%, paO_2_ < 40 mmHg, hypercapnia (paCO_2_ 60 mmHg) and progressive signs of respiratory decompensation (Table 1). During the immediate preparations for reintubation the SpO_2_ degraded further to 35%. Preoxygenation with manual mask-CPAP and inspiratory O2 concentration of 100% (FiO_2_ 1,0) achieved a maximum SpO_2_ of 88%. After i.v. induction (10 mg Midazolam, 100 mg Ketamine, 100 mg Rocuronium) intubation was performed using a video laryngoscope (hyper-angulated blade size 4). Initially the laryngeal view was obstructed by small amounts of a foamy, lightly red-tinged fluid which was removed via suction and the trachea was successfully intubated using a Magill # 8.0 tube. The consecutive bronchoscopy showed no pathological findings. Consecutively an emergency CT scan (thorax, abdomen) was performed (Fig. 1).

**Table 1:**
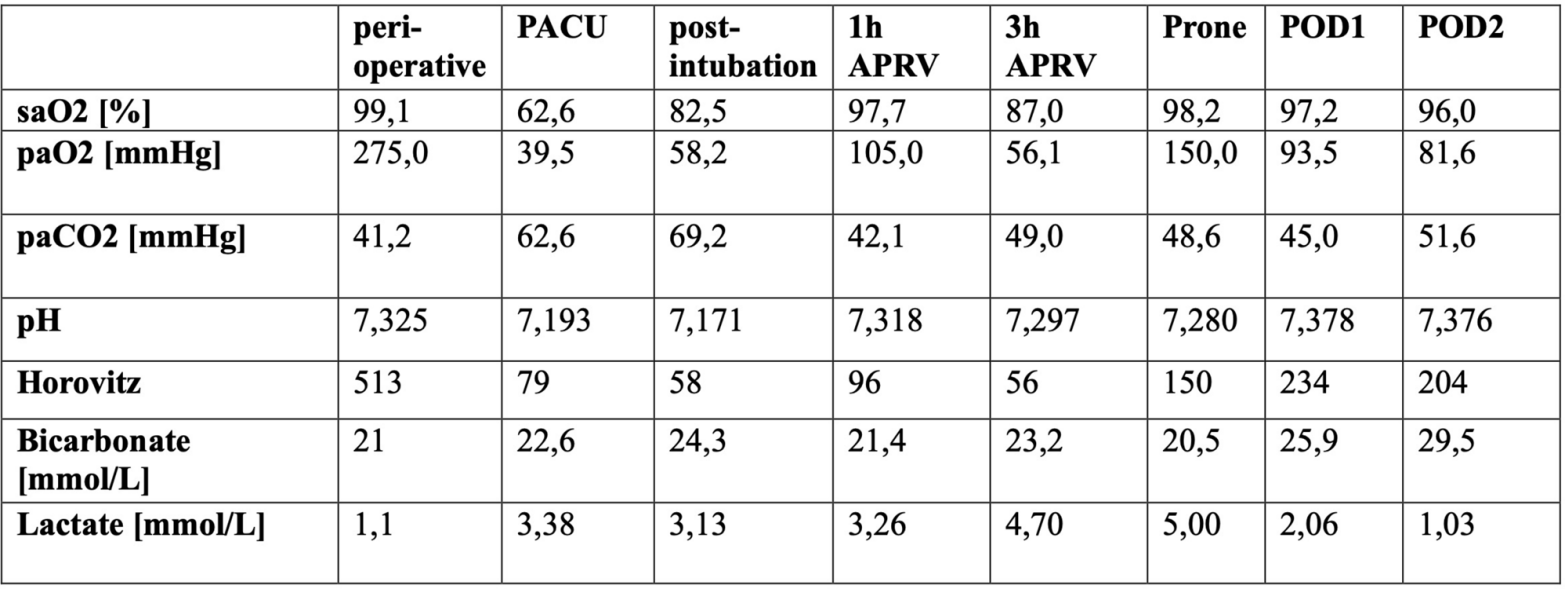
Arterial blood gas parameters from Preoperative, admission to PACU (spontaneously breathing), postintubation on PACU, after one-hour (h) APRV, after 3 h APRV, during prone positioning under APRV, POD1 under APRV, POD2 under APRV. PACV=post anesthesia care unit. APRV=airway pressure release ventilation. POD=post operative day. Values [units]

The CT scan showed massive pulmonary alveolar edema of both lungs, worsening from apical to basal and from anteriorly to posteriorly. After initial stabilization of oxygenation following reintubation, continuous degradation of gas exchange parameters over the next hours was noted. Despite employing a ventilatory strategy using APRV (peak airway pressure 32 mbar, positive end-expiratory pressure 0 mbar, inspiratory to expiratory ratio 7:1, FiO2 1,0) paO2 dropped below 60 mmHg (Table 1). Ultimately the patient rapidly developed the clinical finding of ARDS.

**Fig. 2:**
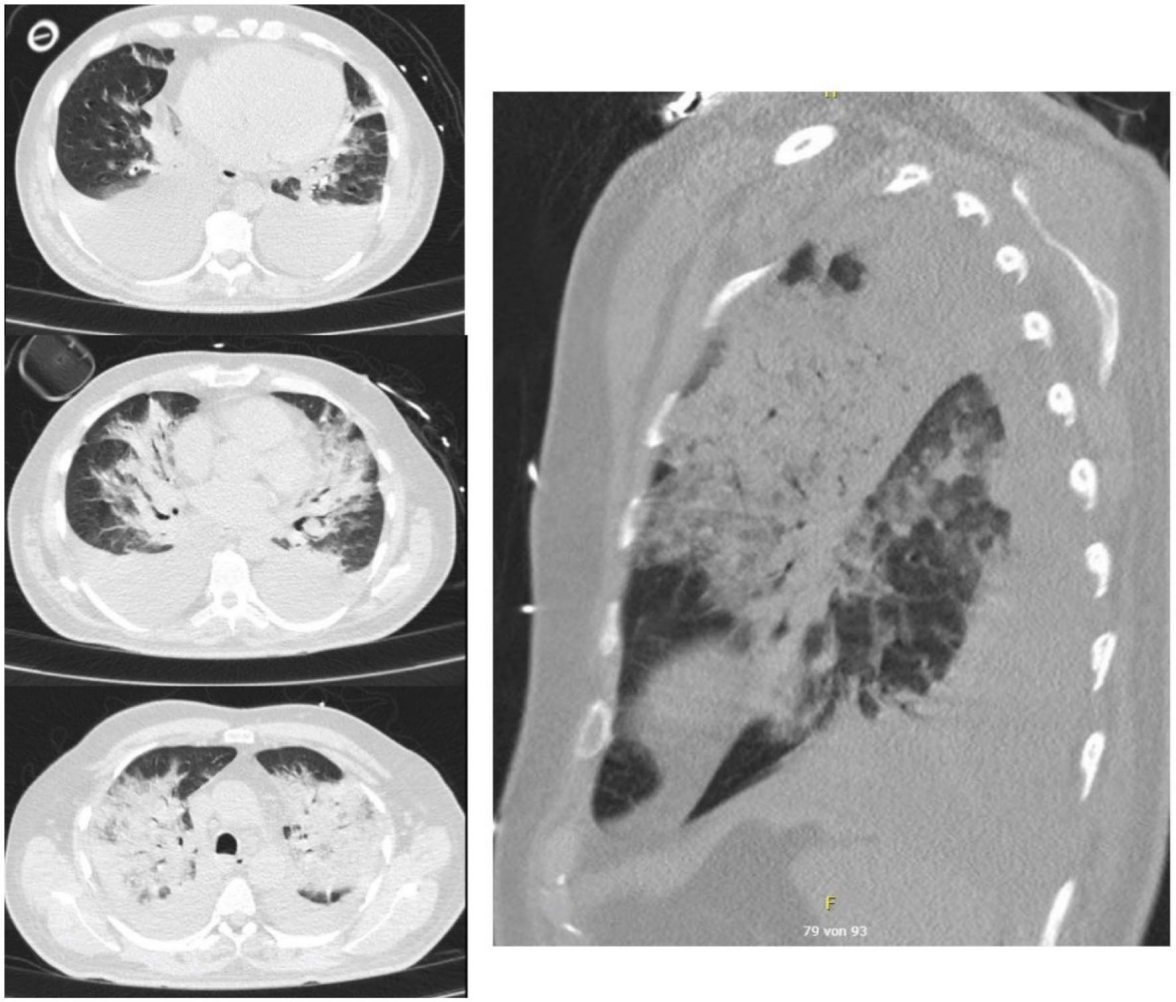
Non-contrasted thoracic CT-scan, postoperative day 7. 3 transversal views from supradiaphragmal to apical (left). 1 sagittal view (right). Progredient consolidations of both upper lobs. Progredient bilateral pleura effusion

In preparation for an eventual extracorporeal pulmonary support the patient was transferred to another in-house intensive care unit. Upon arrival prone positioning was instituted. Approximately 1000 ml of light red fluid was mobilized spontaneously during the prone positioning maneuver and drained from the endotracheal tube indicating pulmonary edema. Following the secrete mobilization oxygenation improved significantly (Horovitz index from 56 to 150). Intermittent prone positioning was terminated after two days. During the next 24 h oxygenation indices continuously improved, leading to a successful extubation on the third postoperative day. In the following period respiration was stabilized using high flow nasal oxygen and non-invasive ventilation. Sonography and a follow-up CT scan on the 7th postoperative day (Fig. 2) showed relevant bilateral pleural effusion which was drained on the 7th and 8th postoperative days.

Echocardiography on postoperative day 10 revealed a possible pulmonary hypertension (systolic pulmonary artery pressure 50 mmHg incl. central venous pressure). Critical care therapy encompassed an empirical i.v antibiotic with Piperacillin/Tazobactam from day 0 to day 6 and Meropenem from day 6 to day 10. The patient was discharged from the intensive care unit on the 10th postoperative day. Despite showing residual symptoms of exertional dyspnea and the feeling of general weakness, the patient was discharged from the hospital on the 14th postoperative day with good cardiopulmonary function.

After discharge written informed consent was obtained from the patient for publication of this case report.

## Discussion and Conclusion

Pulmonary edema can be a symptom or complication of various diseases. The somewhat fragile alveolar architecture is prone to injury, e.g. due to mechanical stress, hydrostatic or hemodynamic changes and/or capillary leakage. Factors contributing to the development of pulmonary edema is low osmotic pressure, high capillary permeability and a high negative pressure gradient and vice versa [1]. The pathophysiological changes ultimately lead to an increase in alveolar diffusion distance, a decrease in gas exchange area and an increased work of breathing.

Negative pressure pulmonary edema (NPPE) is typically caused by a rapid and extreme drop in intrathoracic pressure. Ma et al. report pressures as low as -140 cmH_2_O during forced inspiration [2]. These low intrathoracic pressures may usually occur during spontaneous breathing efforts with an obstructed/occluded airway. Low intrathoracic pressure also transduces to the pulmonary vascular system. As venous return increases pulmonary blood volume and right ventricular workload increase, resulting in an increased transcapillary hydrostatics pressure [2]. Considering hypoxemia is present in many cases of laryngospasm and NPPE, hypoxemic pulmonary vasoconstriction can also contribute to altered capillary permeability. Those mechanisms favor the formation of severe alveolar edema. Furthermore, mechanical stress induced by negative pulmonary pressure increases capillary permeability [2].

Several situations during general anesthesia bear the risk of NPPE. One of the most common settings associated with a high risk for developing a NPPE is the occurrence of a laryngospasm during the post-extubation phase [1, 2]. Furthermore the act of biting from the unconscious patient under general anesthesia onto the endotracheal tube or laryngeal mask can lead to an airway obstruction with a relevant risk of NPPE development [3]. Ma et al. described sleep apnea and anatomical intubation difficulties as individual risk factors for perioperative NPPE [2].

In the non-perioperative setting the disease can occur due to various pathologic changes ranging from severe infections such as epiglottitis or pseudo croup, strangulation injury and vocal cord paralysis due to tumors of the upper airway [3].

Alb et al. describe a type I and type II NPPE. Type I is typically induced by laryngospasm, type II is caused by de-obstruction of chronically obstructed airway. The type II reaction is due to a sudden reduction of chronically enhanced intrinsic PEEP after extubation, e.g. after nasopharyngeal tumor surgery [3].

The therapeutic options for NPPE are focused on treating of the pathophysiological cause. Therefore, positive airway pressures through non-invasive or invasive ventilation are employed to counteract the initial negative transpulmonary pressure and thereby to reduce pulmonary edema [1].

Additional supportive therapy by means of beta-mimetic drug inhalation, steroids and diuretics have been discussed in literature but clear evidence for their benefits is lacking [4, 5].

While the clinical findings in NPPE may impose as a severe case of ARDS, pulmonary dysfunction in most patients resolves within 48 h [5].

Our patient developed an extreme case of NPPE with the rapid degradation of pulmonary function resulting in severe ARDS. Despite the typical mechanical cause of pulmonary edema, anaphylactic capillary disorder might have been a contributing factor in formation of NPPE [6]. After 72 h of invasive ventilatory therapy in conjunction with prone-positioning, successful extubation was achieved. Further respiratory support via high-flow nasal oxygen therapy and non-invasive ventilation was needed until pulmonary recovery. Considering the available literature this case showed a rather severe and prolonged impairment of pulmonary function. This might be due to the suspected multifactorial etiology of NPPE coinciding with an allergic reaction.

Alb et al. suggest a sufficient depth of anesthesia, especially in management of head-neck surgery cases and whenever any airway manipulation under general anesthesia may be warranted. Airway devices might also be protected from biting by various means such as the placement of a Guedel tube [3]. In our case a more delicate termination of anesthesia paired with a higher vigilance for the timepoint of full emergence from general anesthesia prior to extubation might have minimized the risk for NPPE in our patient. However, NPPE may potentially develop in any patient undergoing general anesthesia and should be carefully considered as a possible cause of sudden pulmonary dysfunction occurring after extubation.

“Intubation is a skill, extubation is an art [7]”.

In conclusion negative pressure pulmonary edema is a usually benign, rare, but typical complication of laryngospasm under/after general anesthesia and other clinical scenarios presenting as a severe case of acute respiratory dysfunction occurring after extubation. The pathophysiological mechanisms are complex. Treatment options are aimed at counteracting these underlying mechanisms. Depending on the severity of clinical symptoms invasive ventilation may be necessary for rapid and successful treatment. In most cases the pulmonary dysfunction resolves quickly after initiation of appropriate ventilatory therapy.

## Data Availability

Only the individual data set of clinical routine data of one patient of used in this publication.

## Abbreviations

APRV: Airway pressure release ventilation
ARDS: Acute respiratory distress syndrome
Cm: Centimeter
cmH2O: Centimeter of water
FiO2: Inspiratory Oxygen fraction
i.v.: Intravenous
kg: Kilogram
mmHg: Millimeter of Mercury
NPPE: Negative pressure pulmonary edema
Paco: Arterial partial pressure of Carbon dioxide
Pao2: Arterial partial pressure of Oxygen
PEEP: Positive end-exspiratory pressure
SpO2: Oxygen saturation
TOF: Train-of-Four
